# Patterns and Prognosis of Local Recurrence of Nasopharyngeal Carcinoma after Intensity-modulated Radiotherapy

**DOI:** 10.7150/jca.88148

**Published:** 2024-01-01

**Authors:** Xiao-Tang Xiao, Shi-Qian Zou, Yu-Pei Chen, Rui Guo, Ling-Long Tang, Ying Sun, Jun Ma, Wen-Fei Li

**Affiliations:** State Key Laboratory of Oncology in South China, Guangdong Key Laboratory of Nasopharyngeal Carcinoma Diagnosis and Therapy, Sun Yat-sen University Cancer Center, Guangzhou 510060, P. R. China.

**Keywords:** nasopharyngeal carcinoma, local recurrence, failure pattern, prognosis, IMRT

## Abstract

***Objective*:** To investigate the patterns of local failure and prognosis in patients with locally recurrent nasopharyngeal carcinoma (rNPC) after primary intensity-modulated radiotherapy (IMRT).

***Methods*:** The data of 298 patients with locally rNPC after IMRT were retrospectively analyzed. Magnetic resonance images of the initial and recurrent tumors were reviewed and, for patients with extra-nasopharyngeal local recurrence, the gross tumor volume of local recurrence was transferred to the original IMRT plan for dosimetry analysis. Significant prognostic factors for overall survival (OS) were selected by multivariate Cox regression analysis.

***Results*:** The commonest recurrence sites were the nasopharynx (93%, 277/298) and skull base (53.7%, 160/298). Of the 21 patients with extra-nasopharyngeal recurrence (19 cases valid), 12 had in-field failures, 4 had marginal failures, and 3 had out-field failures. The ethmoid sinus (57.1%, 4/7) and nasal cavity (28.6%, 2/7) were the most frequent sites of marginal and out-field failures. After median follow-up of 37 months, the 3-year and estimated 5-year OS rates were 57.3% and 41.7%, respectively. Multivariate analysis showed that age, recurrence interval, plasma Epstein-Barr virus (EBV) DNA level, and recurrent T stage were independent prognostic factors for OS.

***Conclusions*:** Local failure after IMRT occurs most commonly in the nasopharynx and skull base. In patients with extra-nasopharyngeal recurrence, in-field failure remains the main failure pattern, and marginal and out-field failures mainly occur in the ethmoid sinus and nasal cavity. Elder age, shorter recurrence interval, detectable plasma EBV DNA, and advanced recurrent T stage are negative predictors of OS in patients with rNPC.

## Introduction

Nasopharyngeal carcinoma (NPC) is the most common cancer of the head and neck region in Southeast Asia. It is highly sensitive to radiotherapy and chemotherapy 1. In patients with non-metastatic NPC, intensity-modulated radiotherapy (IMRT) allows delivery of a highly conformal radiation dose to the tumor, with a sharp dose gradient outside the target volume that minimizes irradiation of adjacent organs. Several studies have shown that tumor control and survival are better, and the incidence of severe late adverse events significantly lower, with IMRT than with two-dimensional radiotherapy 2, 3.

Although the application of IMRT and systemic therapy has greatly improved local control of NPC, about 10% of patients still experience local failure 3, 4, mostly in-field failures within the primary tumor volume 5-9. In-field recurrence is usually associated with radiation resistance, whereas marginal and out-field failures are usually due to inadequate target volume delineation and/or dose coverage. Defects in target volume delineation increase the risk of target miss and local recurrence.

In NPC treated with IMRT, local failure sites and patterns are closely related to the quality of target volume delineation. However, because of the low local recurrence rate of NPC, large study samples are difficult to assemble. To our knowledge, this is the largest sample-sized study to analyze the incidence of local recurrence at various anatomic sites and the relationship between local recurrence sites and initial tumor sites in patients with locally rNPC after IMRT. Moreover, we classified local recurrence into nasopharyngeal and extra-nasopharyngeal recurrence. Local recurrence at the nasopharynx is broadly correlated with intrinsic radioresistance, while extra-nasopharyngeal recurrence might be correlated with inadequate radiation dose and/or target volume delineation. Therefore, we focused on cases with extra-nasopharyngeal recurrence, and performed a detailed dosimetric and target volume analysis of the extra-nasopharyngeal recurrence, which has seldom been reported before. The findings of this study may help physicians improve target volume delineation in patients with NPC. We also investigated the outcomes and prognostic factors in this cohort of patients with locally recurrent NPC (rNPC) following salvage treatment to better predict prognosis and guide treatment.

## Methods

### Patients

**Figure 1:**
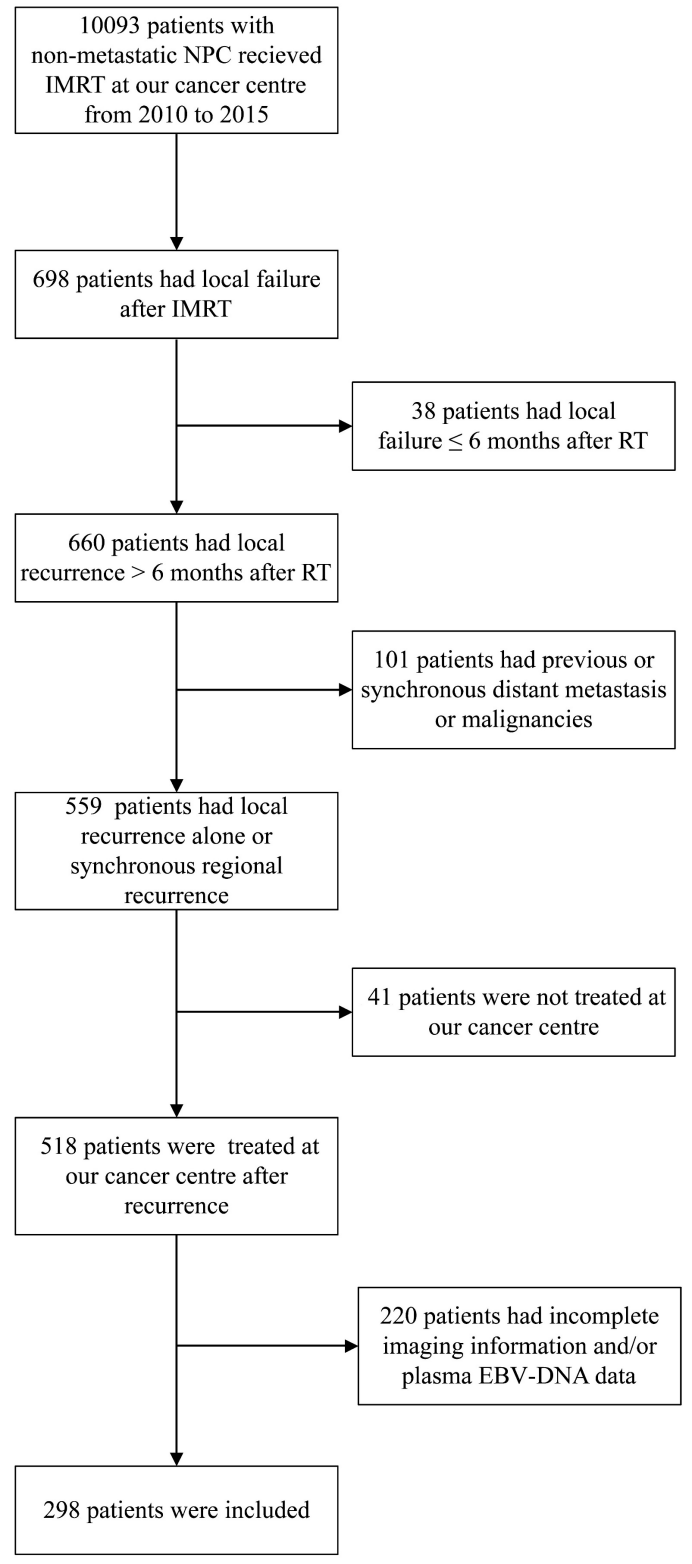
Flow diagram of the study sample selection process (inclusion and exclusion criteria).

The data of patients with newly diagnosed, non-distant metastatic, histologically confirmed NPC treated with IMRT at Sun Yat-sen University Cancer Center from 2010 to 2015 were extracted from the medical records and retrospectively analyzed. The eligibility criteria for inclusion in this study were 1) local or locoregional recurrence occurring > 6 months after the end of primary IMRT and before June 30, 2020; 2) availability of complete imaging data (including magnetic resonance imaging [MRI], computed tomography [CT] and/or 18F-fluorodeoxyglucose positron emission tomography-CT [PET-CT]) of initial and recurrent tumor; 3) no previous or synchronous distant metastasis; 4) no record of partial response to the first course of IMRT; 5) salvage treatments after recurrence included reirradiation, surgery and/or systemic treatment; and 6) no other concomitant malignant tumor or severe comorbidity. The flow chart in Figure 1 shows the patient selection process.

This study was approved by the Institutional Review Board of Sun Yat-sen University Cancer Center, with waiver of the need for informed consent.

### Treatment of initial tumor

All patients underwent radical IMRT with 6-MV photon beam external irradiation. Target volumes and normal tissues were delineated according to our institutional treatment protocol 10. The prescribed doses were 66-72 Gy to the planning target volume of the gross primary tumor volume (GTVp), 60-63 Gy to the high-risk clinical target volume (CTV1), and 54-56 Gy to the low-risk clinical target volume (CTV2), delivered in 28-33 fractions. The irradiation was delivered once daily for 5 days per week. During the study period, our institutional guidelines recommended IMRT alone for stage I disease, and cisplatin-based concurrent chemoradiotherapy with or without neoadjuvant/adjuvant chemotherapy for stage II-IVA disease.

### Diagnosis of local recurrence

**Figure 2:**
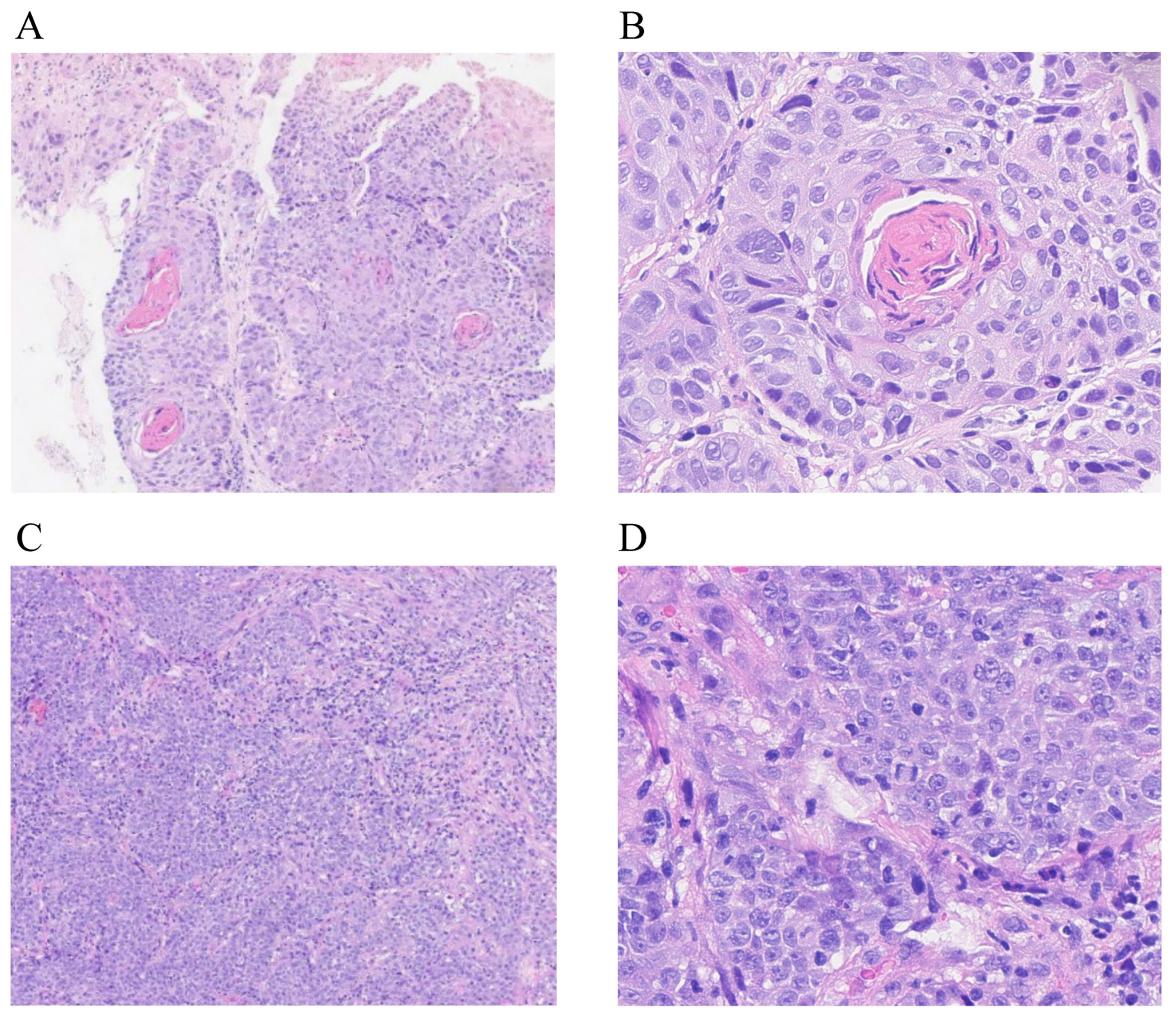
Light microscopic appearance of locally recurrent nasopharyngeal carcinoma. Keratinizing squamous cell carcinoma (A: H&E stain, ×40; B: H&E stain, ×400); non-keratinizing carcinoma, undifferentiated subtype (C: H&E stain, ×40; D: H&E stain, ×400).

Endoscopic or surgical biopsy, or fine-needle aspiration should be used to confirm local or regional recurrence. Eligible patients had histologically confirmed keratinizing squamous, non-keratinizing, or basaloid squamous carcinoma according to the World Health Organization Classification 11 (Figure 2). However, when pathological evidence could not be obtained (e.g., in cases of skull base and intracranial involvement), diagnosis of local recurrence was based on abnormal imaging findings on PET-CT and/or MRI, and/or presence of progressive disease. All imaging-based diagnoses of local recurrence were confirmed retrospectively by two experienced radiologists. Recurrent tumors were staged according to the 8^th^ edition of the Union for International Cancer Control and American Joint Committee on Cancer (UICC/AJCC) staging system 12. Plasma Epstein-Barr virus (EBV) DNA concentrations were routinely measured by quantitative polymerase chain reaction 13. For patients with local recurrence outside the nasopharynx, the gross recurrent tumor volume (GTVr) of the primary site was identified on MRI images and transferred to the original planning CT for dosimetry analysis. The recurrent lesions were evaluated by Dmax, Dmin, Dmean, and volume of recurrent tumor (Vr). The radiation dose received by Vr was calculated and analyzed using dose-volume histograms. Failure patterns were classified as 8, 9: in-field recurrence (i.e., ≥95% Vr within the 95% isodose lines of the primary IMRT plan); 2) marginal-field recurrence (i.e., 20%-<95% Vr within the 95% isodose lines of the primary IMRT plan); or 3) out-field recurrence (i.e., <20% Vr within the 95% isodose lines of the primary IMRT plan).

### Treatment after local recurrence

Salvage locoregional treatments included reirradiation, endoscopic nasopharyngectomy, and neck dissection, alone or in combination. Salvage treatment was determined by the tumor size, extent, and location, as well as by the patient's preference; treatment decisions were made jointly by radiation oncologists and surgeons. The treatment approach can be briefly summarized as follows: 1) reirradiation with IMRT was considered for all locoregional recurrent NPC, but the time interval between the first and second courses of IMRT had to be ≥ 1 year. The GTVr included the primary site and neck, and the CTV included the entire nasopharynx and lymph node-positive regions. The prescribed doses were 60-70 Gy to the GTVr and 50-54 Gy to the CTV, delivered in 27-35 fractions 14, 15; 2) endoscopic nasopharyngectomy was performed for early recurrent disease (rT1-2) or for rT3 disease confined to the floor of the sphenoid sinus. It included endoscopic resection plus microwave ablation, with or without posterior pedicle nasal mucoperiosteal flap resurfacing of the nasopharyngeal defects 16; 3) either radical or selective neck dissection was performed for recurrent neck disease. Chemotherapy and targeted therapy were used in combination with reirradiation or surgery in selected patients.

### Follow-up

After treatment completion, patients were evaluated every 3 months during the first 3 years and every 6 months thereafter until death. Time to recurrence was defined as the time from completion of the primary IMRT to the day of local recurrence. The primary endpoint was overall survival (OS), defined as the time from the day of local recurrence to the date of last follow-up or death.

### Statistical analysis

The McNemar test was used to assess the association between tumor invasion sites in primary NPC and recurrent NPC. Survival rates were estimated using the Kaplan-Meier method, and survival differences were compared by the log-rank test. Multivariate Cox regression analysis 17 with backward elimination was used to identify the independent predictors of survival, and hazard ratios (HRs) and 95% confidence intervals (CIs) were calculated. Statistical analysis was performed using SPSS 26 (IBM Corp., Armonk, NY, USA). Two-tailed P < 0.05 was considered statistically significant.

## Results

### Patient characteristics

Table 1 lists the characteristics of the 298 patients included in this study. The male: female ratio was 2.6:1. Median age at recurrence was 47 years (range, 22-75 years). Diagnosis was confirmed by pathological examination in 250/298 (83.9%) patients. The comparison of histopathological types between primary and recurrent tumors is shown in Table 2. Detectable plasma EBV DNA was present at recurrence in 55% (164/298) patients. The median time to recurrence was 25.8 months (range, 6.4-93.7 months). While, 222/298 (74.5%) patients had local recurrences alone, 76/298 (25.5%) patients had synchronous nodal recurrence. The T stage of recurrent tumor was rT1 in 75/298 (25.2%) patients, rT2 in 33/298 (11.1%) patients, rT3 in 102/298 (34.2%) patients, and rT4 in 88/298 (29.5%) patients. While 103/298 (34.6%) patients were classified as stage I-II, 195/298 (65.4%) were classified as stage III-IVA.

### Patterns of local recurrence

Table 3 shows the distributions of involved sites and the comparisons of the recurrent and initial tumor invasion by McNemar test. The most common recurrence site was the nasopharynx (93%, 277/298), followed by the skull base (53.7%, 160/298), parapharyngeal space (43%, 128/298), prevertebral space (34.9%, 104/298), cavernous sinus (27.9%, 83/298), masticator space (23.2%, 69/298), and the pterygopalatine fossa (22.5%, 67/298). The invasion rates of most sites were lower in rNPC than in primary NPC, the exceptions were cavernous sinus, ethmoid sinus, and orbit.

### Dosimetric analysis of primary IMRT

**Figure 3:**
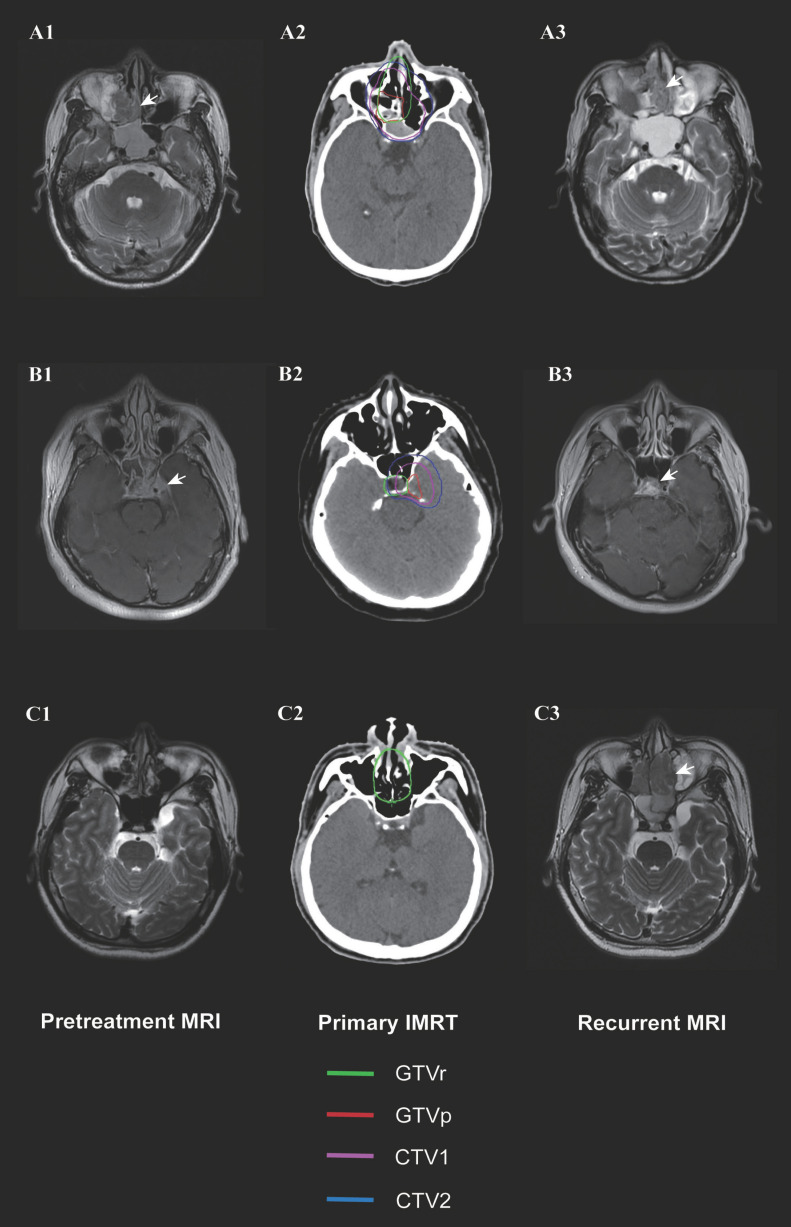
Magnetic resonance imaging (MRI) images of three patients with local recurrence outside the nasopharynx. Marginal failure in the ethmoid sinus and nasal cavity due to the large volume of the recurrent tumor (A1-A3); marginal failure at the hypophyseal fossa due to target miss (B1-B3); and out-field failure in the ethmoid sinus and nasal cavity (C1-C3). Left: Initial diagnostic MRI. Middle: Target volume delineation and dose prescription of primary intensity-modulated radiotherapy. Right: MRI at recurrence. The green line indicates the gross recurrent tumor volume (GTVr); the red line, the gross primary tumor volume (GTVp); the pink line, the high-risk clinical target volume (CTV1); and the blue line, the low-risk clinical target volume (CTV2).

Among the 298 patients, 21 (7%) had extra-nasopharyngeal recurrence; for these patients, we re-located the recurrent tumor in the initial diagnostic imaging and the original IMRT plan for dosimetry analysis. Table 4 shows the clinical characteristics and patterns of local failures of these 21 patients. The original IMRT plans of two patients were lost; of the remaining 19 patients, 12 (63.2%) patients had in-field failures, 4 (21.1%) had marginal failures, and 3 (15.8%) had out-field failures. All were classified as rT3-4. The median doses to the in-field, marginal, and out-field recurrent tumors were 73.41 Gy, 66.14 Gy, and 22.83 Gy, respectively. Among the 12 patients with in-field failures, 10 (83.3%) had skull base invasion, 6 (50%) had cavernous sinus invasion, and 3 (25%) had pterygopalatine fossa invasion. Of the 7 patients with marginal or out-field failures, 4 (57.1%) had recurrence in the ethmoid sinus, 2 (28.6%) in the nasal cavity, and the other recurrent sites included the hypophyseal fossa, orbital apex, sphenoid sinus, frontal sinus, cavernous sinus, and skull base (Figure 3). We further checked whether these sites of marginal and out-field failures were invaded in the pretreatment MRI. The recurrence sites of two patients were invaded in the initial MRI and had been delineated into the GTVp to receive a radical dose; however, the large recurrent tumor volume led to classification as marginal failure. One patient had hypophyseal fossa invasion at initial MRI, but it was not completely included in GTVp; this led to marginal recurrence. The other four patients had recurrent tumors at sites uninvolved at initial imaging.

### Treatment outcome

**Figure 4:**
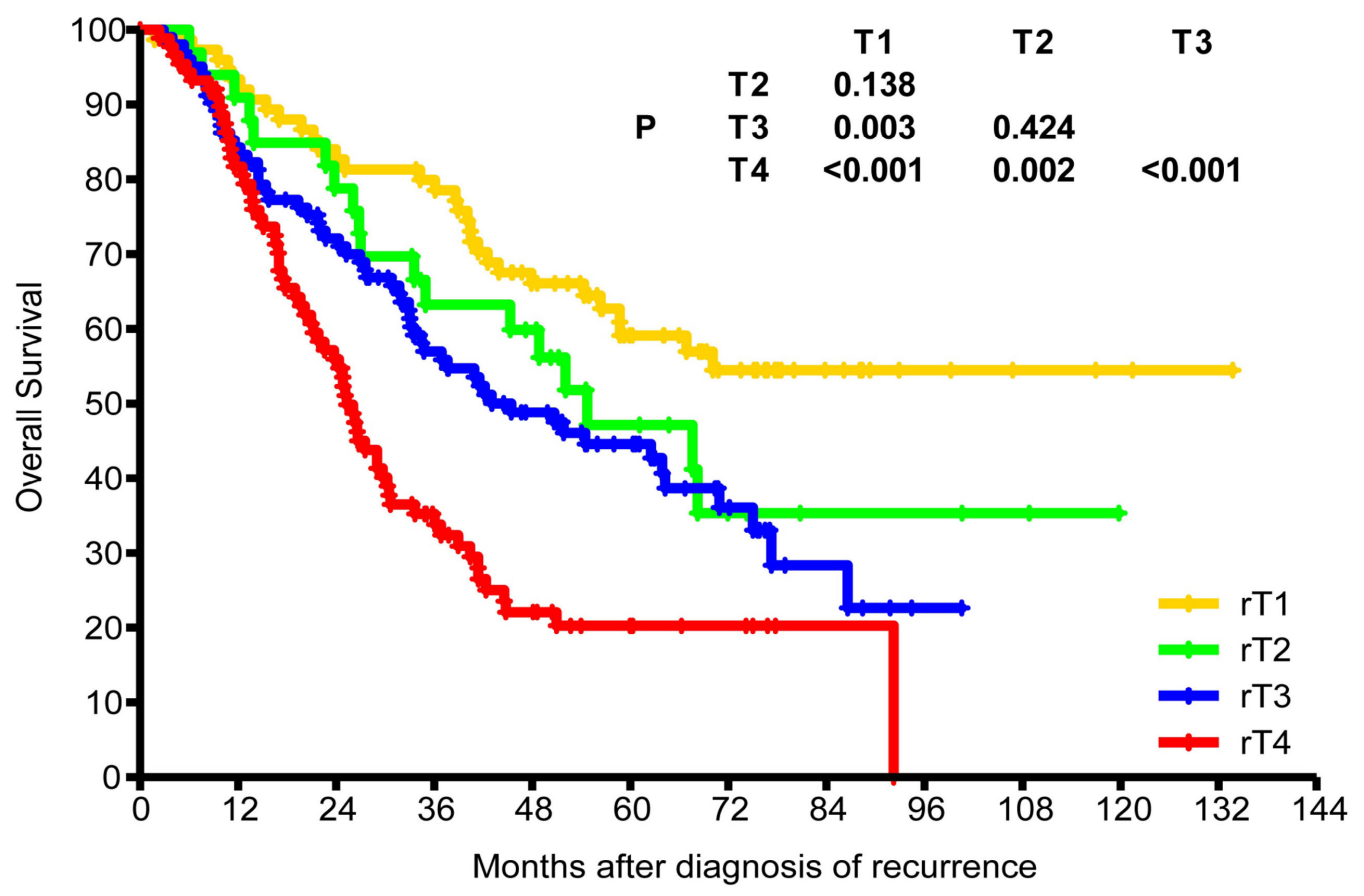
Kaplan-Meier overall survival curves for patients with different recurrent T stages.

Of the 298 patients with recurrence, 65 (21.8%) received surgery alone, 155 (52%) received re-irradiation alone, and 6 (2%) received surgery plus re-irradiation. While 133 (44.6%) patients received local treatment plus chemotherapy, 72 (24.2%) received chemotherapy alone. The median dose of patients who received re-irradiation alone was 62 Gy (range, 24-70 Gy). After median follow-up of 37 months (range, 1.8-133.7 months), the 3-year and estimated 5-year OS rates were 57.3% and 41.7%, respectively. The estimated 5-year OS rates for rT1, rT2, rT3, and rT4 were 59.1%, 47.1%, 44.6%, and 20.3%, respectively (Figure 4). The estimated 5-year OS rates of recurrence stages I, II, III, and IVA were 63.5%, 50.4%, 43.5%, and 19.9%, respectively.

### Univariate and multivariate analyses

Association between OS and sex, age, recurrence interval, plasma EBV DNA level at recurrence, recurrent T stage, recurrence N stage, and treatment after recurrence was examined using univariate and multivariate analyses. Only age, recurrence interval, plasma EBV DNA level, and recurrent T stage were found to be independent prognostic factors for OS (Table 5).

## Discussion

The incidence rates of recurrence at different anatomic sites in patients with locally rNPC have seldom been reported. In the present study, the most common sites of local recurrence were the nasopharynx (93%) and skull base (53.7%), followed by the parapharyngeal space, prevertebral space, cavernous sinus, masticator space, and the pterygopalatine fossa. This is consistent with previous reports 6, 18, 19. In 32 patients with rNPC after IMRT, Li et al. reported recurrence rates of 78.1% and 59.4% in the nasopharynx and skull base, respectively; the authors also found significantly higher invasion rate of the ethmoid sinus in patients with rNPC than in patients with primary NPC 6. In the present study, the invasion rates of cavernous sinus and ethmoid sinus were higher in rNPC than in primary NPC, but the difference was not statistically significant.

Previous studies have found in-field failure to be the main pattern of local recurrence 6-9, 20. Among 710 NPC patients treated with IMRT and followed up for a median of 38 months, Li et al. observed 34 local failures (32 cases valid), of which 16 (50%) were central failures, 9 (28.1%) were marginal failures, and 7 (21.9%) were out-field failures 6. In a retrospective study of 645 patients with new histological-confirmed NPC treated with IMRT and followed up for a median of 62 months, Chen et al. reported 60 cases of locoregional recurrence: 56 (93.3%) in-field failures, 3 (5%) marginal failures, and 1 (1.7%) out-field failure 9. Potential reasons for local recurrence include the following: 1) radiation resistance, i.e., the existence of radio-insensitive stem cells and increase in hypoxic cells 7; 2) large-sized tumors that cannot really be cured 6; 3) intentional restriction of radiation dose because of need for dose limitation to critical organs; and 4) defects in target volume delineation and inaccuracy of radiation treatment plan design 18.

In the present study, we classified local recurrence into two categories: nasopharyngeal and extra-nasopharyngeal recurrence. Inherent resistance to irradiation and/or large tumor size were likely the main reasons for nasopharyngeal recurrence, while inadequate radiation dose and/or target volume delineation might be the main reasons for extra-nasopharyngeal recurrence. Dosimetric analysis of patients with extra-nasopharyngeal recurrence showed that the main recurrence pattern was in-field failure. The ethmoid sinus and nasal cavity were the most common sites of marginal and out-field failures. Further, among the four patients with ethmoid sinus recurrence (all involving the anterior and middle ethmoid sinuses), three patients did not have ethmoid sinus invasion at initial imaging, while one patient had posterior ethmoid sinus involvement. However, two patients had nasal cavity invasion at initial imaging. Thus, concurrent nasal cavity and ethmoid sinus recurrence may be common. According to the international guidelines for CTV delineation for NPC 21, only the posterior-inferior part of the ethmoid sinus is routinely included in the CTV. We suggest that elective coverage of the anterior and middle ethmoid sinuses in patients with nasal cavity involvement might reduce the risk of local recurrence. In our cohort, one patient had hypophyseal fossa invasion at initial MRI, but as this was not completely included in GTVp, the patient developed marginal recurrence. Inaccuracy in GTV delineation that may lead to target miss must be avoided.

Current evidence suggests that surgery should be the treatment of first choice for rNPC, with re-irradiation considered for unresectable disease, and chemotherapy an option to improve long-term survival in patients with advanced rNPC. When formulating individualized treatment for rNPC, the physician should take into consideration the recurrent T stage, tumor size, and condition of patient 22, 23. In this study, the proportion of patients in rT1-2 and rT3-4 were 36.2% and 63.8%, respectively. While 75.8% patients received surgery and/or re-radiotherapy, 24.2% received chemotherapy alone. Most patients receiving chemotherapy alone had larger tumors and/or radiation-related side effects such as radiation encephalopathy, which made re-irradiation inadvisable. The 3-year and estimated 5-year OS rates were 57.3% and 41.7%, respectively. This result is similar to the 3-year and 5-year OS rates of 53.2% and 41.1%, respectively, reported by Tian et al. in a retrospective analysis of 251 patients with local rNPC 14. The poor survival of patients with local rNPC may be attributed to severe irradiation-related complications and/or treatment resistance. New modalities such as hyperfractionated IMRT 24, proton and heavy ion therapy 25, 26, and immunotherapy 27, 28 may improve outcomes in rNPC.

In the present study, multivariate analysis showed that age ≥ 60 years, recurrence interval ≤ 2 years, plasma EBV DNA level > 0 copy/mL, and advanced recurrent T stage were independent predictors of mortality. This result is consistent with previous studies 14, 29-31. Sun et al. found age, hypertension, relapsed T stage, and EBV DNA level to be independent prognostic factors 32. Age-related poor performance status will result in poor tolerance to disease and treatment-related toxicity. Some authors have found worse prognosis in patients with positive pre-retreatment EBV DNA than those with negative EBV DNA 32, 33. Others have shown that longer time to recurrence (>30 months) is associated with better outcomes, probably due to longer tissue recovery time 29. In our cohort, because choice of treatment was related to recurrent T stage, local treatment was significantly associated with better OS in univariate analysis but it was not an independent prognostic factor in multivariate analysis.

This study has several limitations. First, this was a single-center retrospective analysis, and so bias is inevitable. Second, the study included patients treated over a long-time span; there were technological advances and changes in treatment strategies over the study period. Therefore, further research is needed to determine the effect of different treatment regimens on the prognosis of rNPC.

## Conclusion

In patients with nasopharyngeal carcinoma developing local failure after intensity-modulated radiotherapy, the nasopharynx and skull base are the most common sites of recurrence. Among patients with extra-nasopharyngeal local recurrence, in-field failure remains the main failure pattern, and marginal and out-field failures mainly occur in the ethmoid sinus and nasal cavity. Independent negative predictors of overall survival include elder age, shorter recurrence interval, detectable plasma Epstein-Barr virus DNA, and advanced recurrent T stage.

## Figures and Tables

**Table 1:** Patient characteristics (n = 298)

Characteristic	n (%)
**Sex**	
Male	216 (72.5%)
Female	82 (27.5%)
**Age at recurrence**	
< 60 years	247 (82.9%)
≥ 60 years	51 (17.1%)
**Methods for diagnosis of recurrence**	
Pathology	250 (83.9%)
MRI+ PET-CT	36 (12.1%)
MRI	12 (4%)
**EBV DNA level at recurrence**	
0 copy/mL	134 (45%)
> 0 - <4000 copies/mL	119 (39.9%)
≥ 4000 copies/mL	45 (15.1%)
**Recurrence interval**	
≤ 2 years after IMRT	131 (44%)
> 2 years after IMRT	167 (56%)
**Recurrent T stage**	
rT1	75 (25.2%)
rT2	33 (11.1%)
rT3	102 (34.2%)
rT4	88 (29.5%)
**Recurrent N stage**	
rN0	222 (74.5%)
rN1	63 (21.1%)
rN2	12 (4%)
rN3	1 (0.3%)
**Recurrent clinical stage**	
rI	55 (18.5%)
rII	48 (16.1%)
rIII	106 (35.6%)
rIVA	89 (29.9%)
**Treatment after recurrence**	
Surgery and/or reirradiation alone	93 (31.2%)
Chemotherapy alone	72 (24.2%)
Surgery and/or reirradiation + chemotherapy	133 (44.6%)

Abbreviations: EBV: Epstein-Barr virus; IMRT: intensity-modulated radiotherapy; MRI: magnetic resonance imaging; PET: positron emission tomography; CT: computed tomography.

**Table 2:** Comparison of histopathological types between pNPC and rNPC (n = 298)

Histopathological Types	pNPC, n (%)	rNPC, n (%)
Keratinizing squamous cell carcinoma	7 (2.3%)	5 (1.7%)
Non-keratinizing carcinoma	291 (97.7%)	245 (82.2%)
Unknown	0 (0%)	48 (16.1%)

Abbreviations: pNPC = primary nasopharyngeal carcinoma; rNPC = recurrent nasopharyngeal carcinoma.

**Table 3:** Comparison of tumor invasion in pNPC and rNPC (n = 298)

Tumor invasion	rNPC, n (%)	pNPC, n (%)	pNPC and rNPC, n	Isolated invasion in pNPC, n	Isolated invasion in rNPC, n	No invasion in pNPC or rNPC, n	P
**Nasopharynx**	277 (93%)	298 (100%)	277	21	0	0	<0.001
**Skull base**	160 (53.7%)	232 (77.9%)	129	103	31	35	<0.001
**Parapharyngeal space**	128 (43%)	271 (90.9%)	120	151	8	19	<0.001
**Prevertebral space**	104 (34.9%)	182 (61.1%)	74	108	30	86	<0.001
**Cavernous sinus**	83 (27.9%)	79 (26.5%)	40	39	43	176	0.741
**Masticator space**	69 (23.2%)	91 (30.5%)	27	64	42	165	0.041
**Pterygopalatine fossa**	67 (22.5%)	90 (30.2%)	40	50	27	181	0.012
**Nasal cavity**	52 (17.4%)	107 (35.9%)	28	79	24	167	<0.001
**Sphenoid sinus**	52 (17.4%)	65 (21.8%)	29	36	23	210	0.117
**Ethmoid sinus**	21 (7%)	19 (6.4%)	6	13	15	264	0.851
**Oropharynx**	17 (5.7%)	33 (11.1%)	4	29	13	252	0.020
**Orbit**	12 (4%)	12 (4%)	5	7	7	279	1.000

Abbreviations: rNPC: recurrent nasopharyngeal carcinoma; pNPC: primary nasopharyngeal carcinoma.

**Table 4:** Clinical characteristics and patterns of local failures of the 21 patients with extra-nasopharyngeal recurrence

No. of patient	Age at recurrence (years)	Sex	Stage	Recurrence site	DVH Statistics to recurrence volume				Type of relapse
					Dmean (Gy)	Dmin (Gy)	Dmax (Gy)	Tumor volume (cm^3^)	
1	44	Male	T4N0M0 IVA	Petrous apex, prepontine cistern	73.78	71.81	76.46	2.90	In-field
2	24	Male	T4N0M0 IVA	Parapharyngeal space, cavernous sinus	73.91	70.19	77.37	23.80	In-field
3	47	Male	T4N0M0 IVA	Cavernous sinus	72.94	66.47	77.22	3.80	In-field
4	47	Female	T4N0M0 IVA	Pterygopalatine fossa, skull base, cavernous sinus	74.32	70.35	77.60	8.80	In-field
5	53	Male	T4N0M0 IVA	Pterygopalatine fossa, skull base, cavernous sinus	75.25	71.47	78.17	34.00	In-field
6	37	Male	T3N0M0 III	Prevertebral space, occipital clivus	70.73	67.25	75.11	11.80	In-field
7	48	Male	T4N0M0 IVA	Foramen ovale, cavernous sinus	72.99	70.63	75.39	2.90	In-field
8	62	Female	T3N0M0 III	Skull base, sphenoid sinus, pterygopalatine fossa	74.47	70.73	76.97	6.40	In-field
9	50	Female	T3N0M0 III	Pterygoid process	72.84	70.53	75.53	1.70	In-field
10	37	Male	T3N0M0 III	Skull base	71.72	63.42	77.74	5.51	In-field
11	67	Male	T4N1M0 IVA	Skull base, cavernous sinus, prepontine cistern	73.75	66.79	78.60	44.80	In-field
12	60	Male	T3N0M0 III	Prevertebral muscle, skull base	73.07	63.92	76.97	22.80	In-field
13	68	Male	T4N0M0 IVA	Skull base, cavernous sinus	74.67	44.47	80.65	32.30	Marginal
14	63	Male	T3N0M0 III	Nasal cavity, ethmoid sinus	69.97	51.80	78.02	40.36	Marginal
15	50	Male	T4N0M0 IVA	Pituitary, hypophyseal fossa	62.31	50.83	74.53	4.12	Marginal
16	49	Male	T4N0M0 IVA	Sphenoid sinus, orbital apex	61.50	12.13	79.60	23.86	Marginal
17	31	Female	T3N0M0 III	Ethmoid sinus	41.43	6.33	64.09	5.00	Outside
18	43	Male	T3N0M0 III	Nasal cavity, ethmoid sinus	22.83	2.95	63.05	39.20	Outside
19	30	Male	T3N0M0 III	Ethmoid sinus, frontal sinus	12.94	3.10	46.06	3.30	Outside
20	54	Male	T4N0M0 IVA	Pterygopalatine fossa, cavernous sinus	/	/	/	/	/
21	55	Male	T4N0M0 IVA	Trigeminal nerves, cavernous sinus	/	/	/	/	/

Abbreviations: DVH: dose-volume histogram.

**Table 5:** Univariate and multivariate analyses of prognostic factors for overall survival

Variable	Univariate analysis			Multivariate analysis		
	HR	95% CI	P	HR	95% CI	P
**Sex female vs. male**	0.92	0.66-1.28	0.604	1.05	0.74-1.49	0.792
**Age at recurrence** ≥ 60 vs. < 60 years	1.83	1.28-2.63	0.001	1.86	1.29-2.68	0.001
**Recurrence interval** > vs. ≤ 2 years after IMRT	0.75	0.55-1.01	0.057	0.64	0.47-0.87	0.004
**EBV DNA level**			<0.001			0.012
> 0 to < 4000 vs. 0 copies/mL	1.64	1.17-2.30	0.004	1.52	1.07-2.15	0.018
≥ 4000 vs. 0 copies/mL	2.31	1.53-3.50	<0.001	1.79	1.17-2.73	0.007
**Recurrent T stage**			<0.001			<0.001
rT2 vs. rT1	1.52	0.85-2.72	0.157	1.77	0.98-3.18	0.058
rT3 vs. rT1	1.91	1.23-2.95	0.004	1.79	1.15-2.79	0.011
rT4 vs. rT1	3.47	2.25-5.37	<0.001	3.28	2.11-5.10	<0.001
**Recurrent N stage**			0.127			0.127
rN1 vs. rN0	1.41	1.00-2.01	0.053	1.47	1.01-2.12	0.042
rN2-3 vs. rN0	1.35	0.69-2.66	0.387	1.04	0.52-2.07	0.908
**Surgery and/or reirradiation** yes vs. no	0.55	0.39-0.76	<0.001	0.79	0.56-1.11	0.168

Abbreviations: HR: hazard ratio; CI: confidence interval; IMRT: intensity-modulated radiotherapy; EBV: Epstein-Barr virus.

## Abbreviations

CI: confidence interval
CT: computed tomography
CTV1: high-risk clinical target volume
CTV2: low-risk clinical target volume
EBV: Epstein-Barr virus
GTVp: gross primary tumor volume
GTVr: gross recurrent tumor volume
HR: hazard ratio
IMRT: intensity-modulated radiotherapy
MRI: magnetic resonance imaging
NPC: nasopharyngeal carcinoma
OS: overall survival
PET-CT: positron emission tomography-computed tomography
rNPC: recurrent nasopharyngeal carcinoma
Vr: volume of recurrent tumor
